# Unusual Presentation of Familial Unilateral Postaxial Polydactyly With Metacarpal Delta Phalanx: A Report of a Rare Case

**DOI:** 10.7759/cureus.49484

**Published:** 2023-11-27

**Authors:** Rakan H Alelyani, Othillah M Moazin, Hana A Alazzmi, Emad A Alfadhel, Hussain Alobaidi, Sultan Alaqil, Tanveer A Bhat, Eyad Nawwab, Mohammed Y Mirza

**Affiliations:** 1 Department of Plastic and Reconstructive Surgery, King Saud Medical City, Riyadh, SAU

**Keywords:** associated anomaly, plastic and reconstructive surgery, rare variant, post-axial polydactyly, delta phalanx

## Abstract

The term "delta phalanx" is proposed to characterize an uncommon deformity that typically affects the middle phalanx of a finger. It has the appearance of the Greek capital letter delta, meaning it is shaped like a triangle. Because the faulty epiphysis occurs proximally to distally instead of along its usual horizontal course, the bone has a semilunar shape. Functional impairment or significant finger shortening are indications for surgery. A variety of congenital hand anomalies are linked to the delta phalanx. Few cases of middle delta phalanx in the ulnar polydactyly finger have been documented. This case study investigates an extremely uncommon occurrence of metacarpal delta phalanx in an ulnar polydactyly finger.

## Introduction

The term "delta phalanx" is suggested to describe a rare deformity, usually occurring in the middle phalanx of a finger, in which the bone is triangular in shape and has a continuous epiphysis running from the proximal to the distal end along the shortened side [1]. This congenital anomaly was first described in 1964 by Jones, who reported five male patients with the same condition [2]. It resembles a triangular-shaped Greek capital letter delta. The bone has a semilunar shape because the faulty epiphysis runs proximally to distally rather than in its typical horizontal course [3]. The delta phalanx has been described in hands as well as in feet. When present in the hands, it is usually associated with an angulation of the digit, such as in clinodactyly, or interposed as an extra phalanx in a triphalangeal thumb [4]. Surgical indications are functional impairment and/or considerable shortening of the finger. Osteotomies are the primary surgical option: opening wedge osteotomy, closing wedge osteotomy, or reverse osteotomy [5].

The delta phalanx can be associated with different congenital hand anomalies. There were minimal reports regarding the occurrence of the middle delta phalanx in the ulnar polydactyly finger. However, such a lesion should raise suspicion of other anomalies. Africans and African Americans are more likely to have postaxial polydactyly. Bilateral polydactyly is possible. While in Caucasians, preaxial polydactyly is more common. One in 143 live births is thought to have postaxial polydactyly among African Americans [6]. By contrast, one in every 1,339 live births in the Caucasian population is thought to have postaxial polydactyly. In Caucasian individuals, postaxial polydactyly may occasionally be a sign of an underlying syndrome, such as chondroectodermal dysplasia or Ellis-van Creveld syndrome [7]. Therapeutic options are known and standardized. Simple monitoring is advised if appearance is the only complaint.

Our case examines a rare incidence of metacarpal delta phalanx in an ulnar polydactyly finger, which is a very rare presentation.

## Case presentation

A 24-year-old female attended our outpatient department (OPD) with a history of a deformed extra finger on the outside of the little finger of her right hand present since birth. Otherwise, she was in normal health. This extra finger was causing difficulty in her activities of daily life (ADL) as it was misaligned and deformed in shape, besides leading to aesthetic deformity of the hand. The other hand and both feet of the patient had a normal number of digits. The parents of the patient were in a consanguineous marriage and had eight kids, our patient was born after full-term pregnancy and was sixth in birth order among the siblings. Her father and two brothers also have an extra finger in their single hands. The general physical examination of the patient was grossly normal, with no apparent other associated anomalies. On local examination of the hand, there was an extra digit on the ulnar aspect of the little finger arising from the metacarpophalangeal (MCP) joint. The extra digit was deformed with a decreased range of movements (ROM) and was relatively shorter than the normal fingers, with its long axis perpendicular to the long axis of the hand. The other digits were normal in size, shape, form, and function.

**Figure 1:**
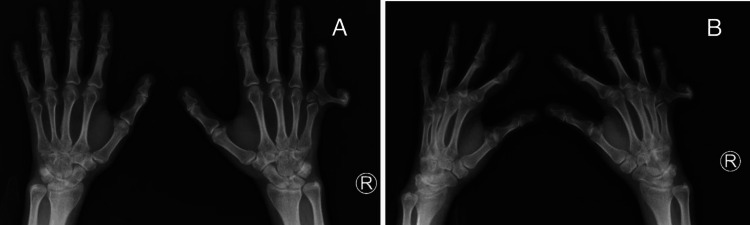
X-ray images of both hands of the patient show type 2 post-axial polydactyly with delta phalanx of the right hand; A. anteroposterior view; B. oblique view

Figure 1 shows an anteroposterior (AP) and an oblique X-ray view of both hands having complete duplication of the little finger of the right hand (type 2 Stelling and Turek’s classification postaxial polydactyly) with a common metacarpal and the presence of a metacarpal delta phalanx articulating distally with the bases of the proximal phalanges of the fifth and the extra digit and proximally with the single fifth metacarpal head.

**Figure 2:**
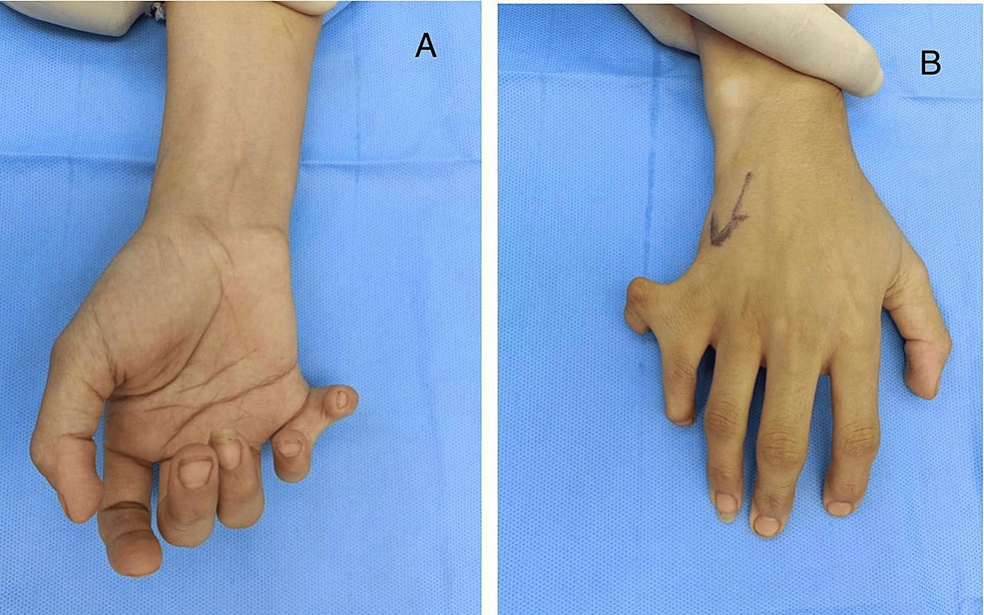
A preoperative photograph of the patient with postaxial polydactyly of the right hand; A. volar aspect of the right hand; B. dorsal aspect of the right hand

**Figure 3:**
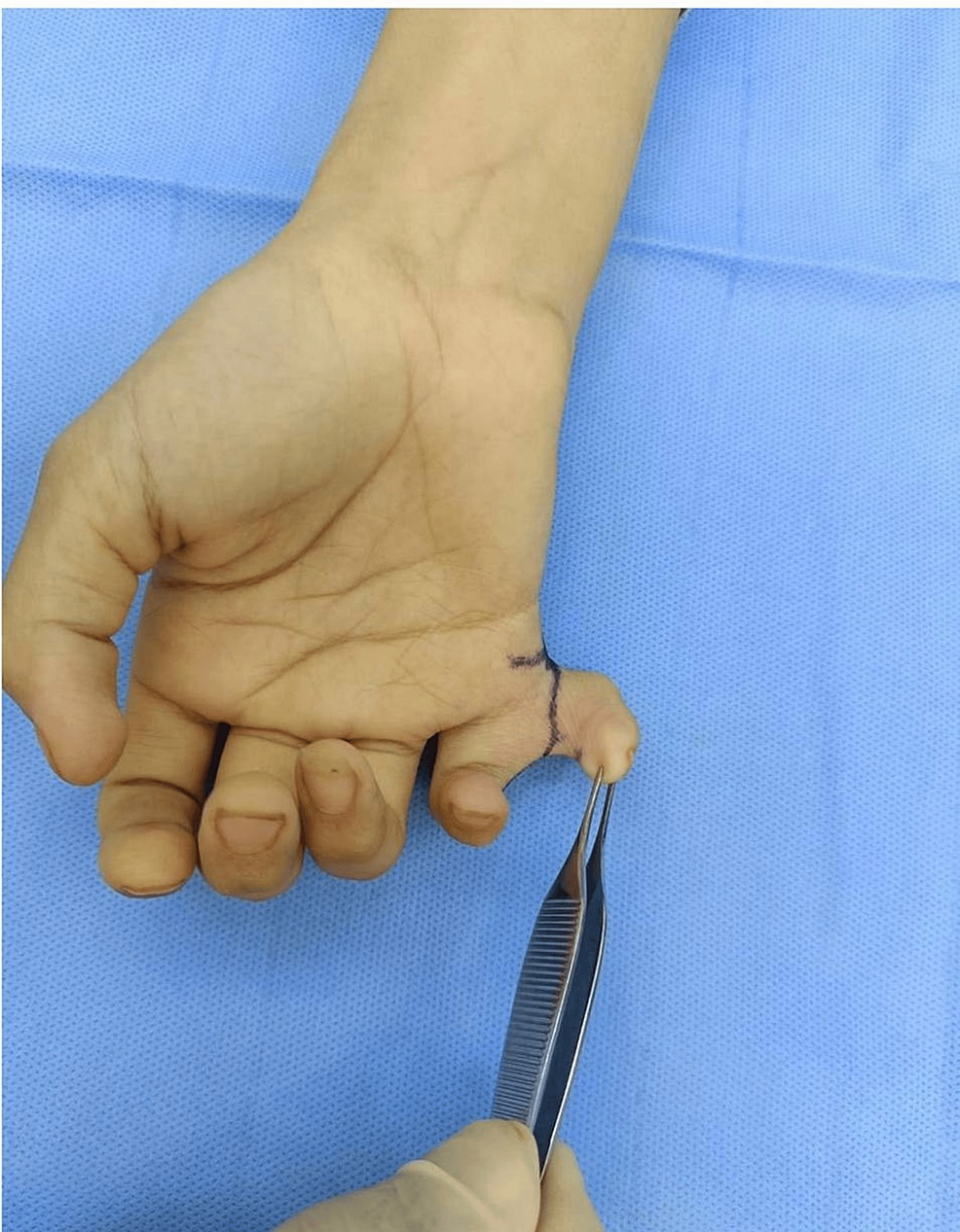
Marking of the incision for the excision of the extra digit in the right hand

The patient was admitted for daycare surgery, and after explaining the treatment plan to her, informed consent was obtained, and the patient was prepared for surgery. Baseline investigations, including a complete blood count (CBC) and kidney and liver function tests, were advised. The patient was subjected to regional anesthesia and operated under tourniquet control with a pressure of 250 mmHg for 45 minutes. After preparing the operative part, a double racquet-shaped incision was marked at the base of the extra digit, planned in such a way that the final scar lay mid-axially on the little finger (Figures 2-3).

**Figure 4:**
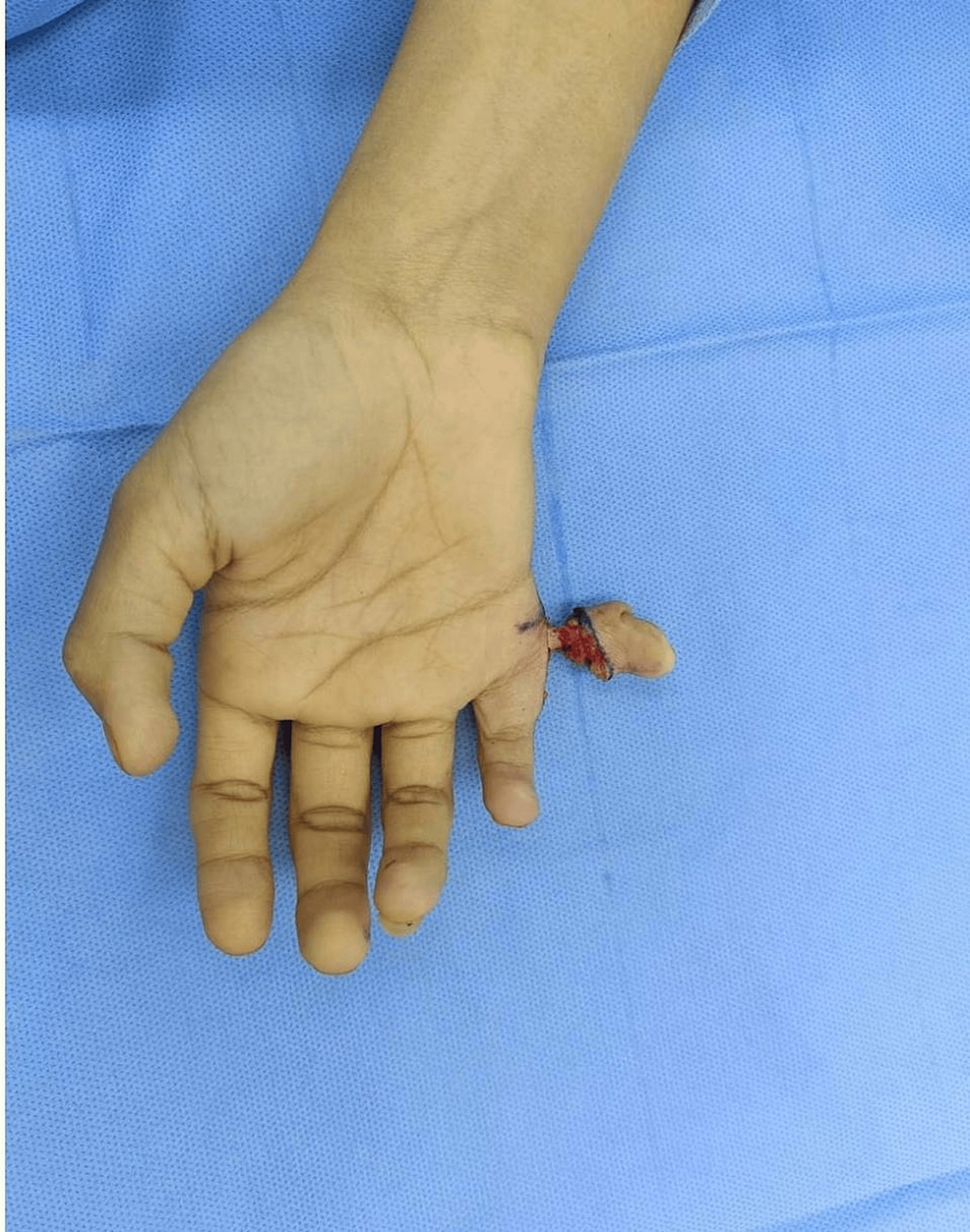
An intraoperative picture showing the soft tissue dissection for the excision of the extra digit

An incision was made and carried through the skin and subcutaneous tissue. A sharp dissection was done using tenotomy scissors. The tendons were found to be attenuated, and we divided these tendons after ensuring that the little finger carries healthy tendons with itself. The digital nerve was dissected, pulled distally, and divided using cautery, and then the cut ends were buried under the soft tissue to avoid painful neuroma formation (Figure 4).

**Figure 5:**
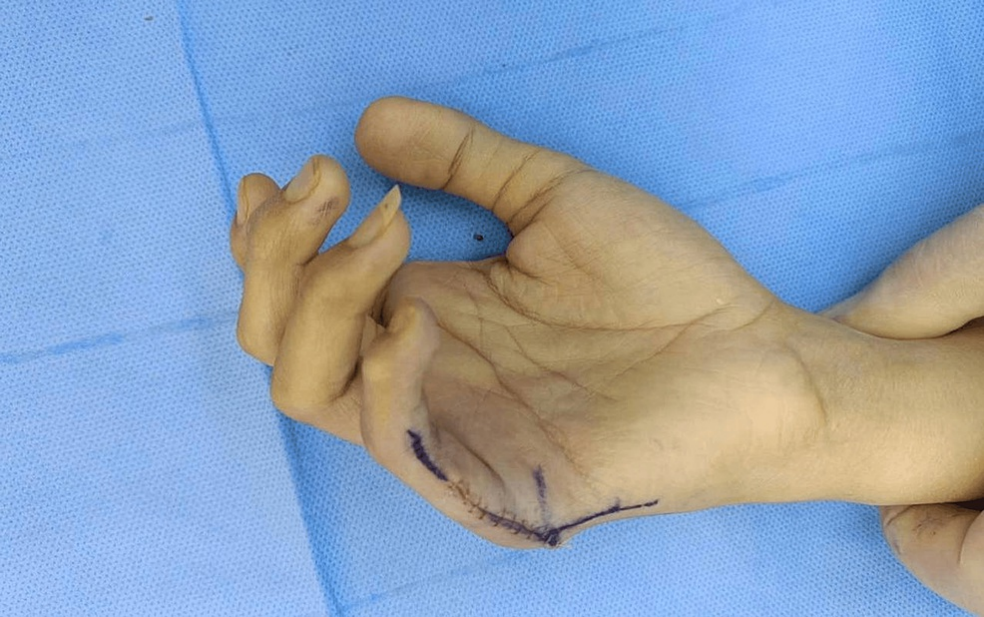
An intraoperative image showing the closure of the incision after the excision of the extra digit

After periosteal ligamental flap elevation, under C-arm guidance, excision of the extra digit was done using a small osteotome, ensuring there was adequate periarticular bony stock to maintain the MCP joint stability. The osteotomy site was filed using a small filer to ensure it was smooth and there were no bony spikes. Repair of the ulnar collateral ligament and the joint capsule was done using 3-0 Prolene, and the stability of the joint was checked following the repair. The torch was deflated, hemostasis was achieved, and the wound was closed in two layers (Figure 5).

**Figure 6:**
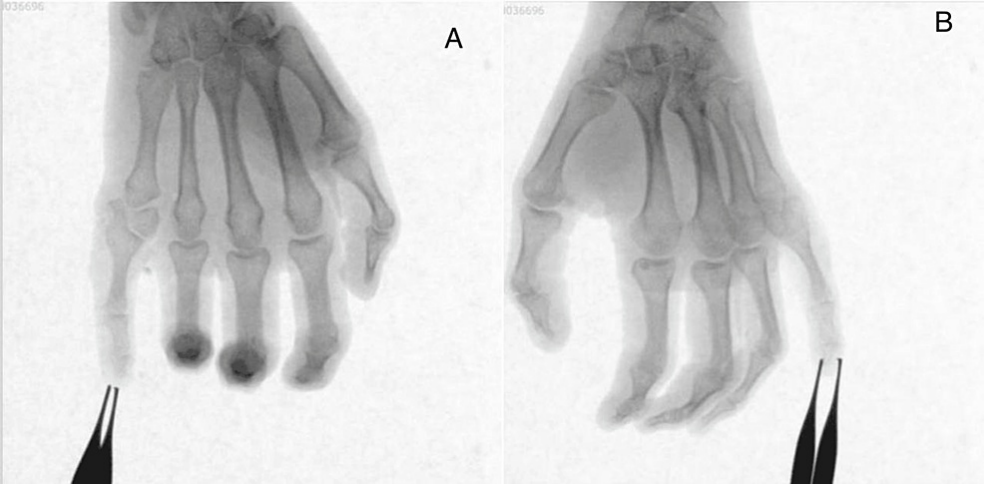
A postoperative X-ray image of the right hand of the patient after excision of the extra digit; A. anteroposterior view of the right-hand; B. oblique view of the right-hand

The dressing was done, and a volar slab in the functional position of the hand was applied. The patient was put on oral antibiotics and analgesics, discharged the same day after full recovery and advised to follow up on OPD after one week. The slab was removed after two weeks on the next follow-up visit (Figure 6).

## Discussion

This report describes a rare instance of familial unilateral ulnar polydactyly type A with metacarpal delta phalanx. As far as we are aware, no prior reports of metacarpal delta phalanges in the case of an ulnar polydactyly exist. The combination of abnormal bone formation, duplication, and failure of differentiation results in this complex congenital anomaly. Polydactyly may occur concurrently with delta phalanges. The delta phalanx was considered by Watson and Boyes to be a typical example of polydactylism [8].

Polydactyly present as unilateral or bilateral and can affect any limb [9,10]. Polydactyly can be categorized as postaxial, preaxial, or central based on where the duplicate digits are located radioulnarly. In the Middle East, the incidence of unilateral polydactyly is much more common as opposed to bilateral polydactyly, with a slight prevalence among women [9]. Moreover, while type A supernumerary digit, which is a well-developed extra digit, occurs in the Middle Eastern population, type B, which is a rudimentary and pedunculated extra digit, occurs more frequently among Africans and Caucasians [11]. The diagnosis of polydactyly is usually clinical, but the use of radiographs taken from different perspectives can accurately classify this entity as an anomaly.

Jones was the first to describe delta phalanges, which are abnormally shortened hands and feet bones. The defective epiphyses, which run the length of the phalanx rather than resting at its ends, are what cause the aberrant morphology. Despite the fact that a cartilaginous bracket is present from birth, the affected bone has not grown enough to show the bracket. Therefore, until the child is two years old, this anomaly is not visible on plain film [3]. The affected finger gradually becomes shorter and more angular due to the obstruction of normal longitudinal digit growth caused by a triangular bone with a C-shaped epiphysis extending along the shorter side of the phalanx [12,13]. The aberrant arrangement is thought to be the result of a primary ossification center defect that occurred during the embryonic stage [14]. The surgical treatment of the delta phalanx was previously advised to involve several types of osteotomies, such as open wedge osteotomies, close wedge osteotomies, and reverse wedge osteotomies with or without bone grafts. These techniques, however, produced unsatisfactory results because, in the majority of cases, osteotomy with recurrence of the deviation followed the early closure of the epiphyseal growth plate and formation of the bone bridge [15].

Despite the fact that our patient didn’t have angulation or clinodactyly of the little finger, it is crucial to understand this entity of anomaly as it is very common with the delta phalanx. Digit angulation in the radioulnar plane is very common in the general population, and deviations of up to 10° are considered normal. Most of these cases are not functionally limiting, and most can be treated conservatively. Surgical intervention may be necessary, though, in cases involving more significant deformities because of the potential functional and aesthetic consequences. In general, an angulation of 25° or more is typically regarded as an indication that surgery is required to avoid functional problems [15].

Usually, delta phalanges involve the middle phalanx, though there are a few reported cases of proximal delta phalanx. Because of the proximal location of the lesion, these frequently show more significant angulation at an earlier age and have a higher chance of resulting in functional disability. Interestingly, the patient had a normal angle and gross functional range of motion. Because of these factors, it was decided that the best course of action for correction would be to do operative separation of the extra digit with the transfer of important parts and to leave the delta phalanx in its space.

## Conclusions

The delta phalanx is usually present on the middle phalanx of the ulnar clinodactyly. However, this aberration can manifest with other types of congenital hand anomalies, like polydactyly, as is seen in our patient with the metacarpal delta phalanx of the extra digit, which is probably the first such case to be reported. The surgical management of each case should be individualized according to the circumstances and primary outcome.
